# Crystal Structure of the FAD-Containing Ferredoxin-NADP^**+**^ Reductase from the Plant Pathogen *Xanthomonas axonopodis* pv. citri

**DOI:** 10.1155/2013/906572

**Published:** 2013-08-01

**Authors:** María Laura Tondo, Ramon Hurtado-Guerrero, Eduardo A. Ceccarelli, Milagros Medina, Elena G. Orellano, Marta Martínez-Júlvez

**Affiliations:** ^1^Molecular Biology Division, Instituto de Biología Molecular y Celular de Rosario (IBR), CONICET, Facultad de Ciencias Bioquímicas y Farmacéuticas, Universidad Nacional de Rosario, Suipacha 531, S2002LRK Rosario, Argentina; ^2^Fundación ARAID, Edificio Pignatelli 36, 50004 Zaragoza, Spain; ^3^Institute of Biocomputation and Physics of Complex Systems (BIFI), Joint Unit BIFI-Rocasolano, Universidad de Zaragoza, 50018 Zaragoza, Spain; ^4^Departamento de Bioquímica y Biología Molecular y Celular, Facultad de Ciencias, Pedro Cerbuna 12, Universidad de Zaragoza, 50009 Zaragoza, Spain

## Abstract

We have solved the structure of ferredoxin-NADP(H) reductase, FPR, from the plant pathogen *Xanthomonas axonopodis* pv. citri, responsible for citrus canker, at a resolution of 1.5 Å. This structure reveals differences in the mobility of specific loops when compared to other FPRs, probably unrelated to the hydride transfer process, which contributes to explaining the structural and functional divergence between the subclass I FPRs. Interactions of the C-terminus of the enzyme with the phosphoadenosine of the cofactor FAD limit its mobility, thus affecting the entrance of nicotinamide into the active site. This structure opens the possibility of rationally designing drugs against the *X. axonopodis* pv. citri phytopathogen.

## 1. Introduction

Ferredoxin-NADP(H) reductases (FNRs, EC 1.18.1.2) constitute a family of hydrophilic FAD-containing monomeric enzymes that deliver NADPH or low potential one-electron donors to redox-based metabolisms in plastids, mitochondria, and bacteria [1]. In heterotrophic bacteria, this activity provides reduced ferredoxin and flavodoxin to diverse reactions involved in amino acid and nucleotide metabolisms, biotin synthesis, and iron-sulphur cluster assembly, as well as for the nitrogenase complex [1]. Based on phylogenetic analysis, the FNR forms present in most prokaryotes (collectively known as FPRs) have been classified into two subclasses represented by the *Azotobacter vinelandii* (subclass I) and the *Escherichia coli* (subclass II) FPR prototypes [1]. Structures of bacterial FPRs, as well as those of plastidic FNRs, are folded in two distinct domains: the C-terminus with a binding site for NADP(H) and the N-terminus binding the cofactor FAD. Unlike plastidic enzymes, bacterial ones show a bent conformation of FAD. This conformation is stabilised by an intramolecular H-bond between the N6 atom of the adenosine and the N1 atom of the isoalloxazine rings, and by stacking of an aromatic side chain against the carboxy-terminal extension and the adenosine moiety. The very low turnover rates for the oxidation of NADPH exhibited by bacterial FPRs in comparison with plastidic enzymes may be associated with this FAD folded conformation, unlike the extended conformation in plastidic FNRs [2] (see Table S1 in Supplementry Material available online http://dx.doi.org/10.1155/2013/906572).

*Xanthomonas axonopodis* pv. citri is a gram-negative bacterium responsible for citrus canker, a severe disease that affects most commercial citrus crops. Bacteria penetrate host plant tissues through stomata and wounds and colonize the apoplast causing cell hyperplasia. Symptoms are ultimately visualized as necrotic corky lesions on leaves, stems, and fruit surfaces. This disease has an important economic impact worldwide, due to reduction of fruit quality, premature abscission of fruits and leaves, and general tree decline [3]. The genome of *X. axonopodis* pv. citri contains the *fpr* gene that encodes the FPR (*Xac*FPR). Despite the fact that neither the role of *Xac*FPR nor its potential substrate has been elucidated, its involvement in the oxidative stress response of *X. axonopodis *pv. citri via interaction with ferredoxin XAC1762 has been proposed [4]. Therefore, *Xac*FPR appears to be a key enzyme for pathogen survival, and the inhibition of its activity might represent an effective treatment against citrus canker caused by *X. axonopodis* pv. citri. 

*Xac*FPR possesses characteristic features of subclass I bacterial reductases, but its functional characterization revealed differences in the isoalloxazine environment related to the subclass I bacterial FPR from *Rhodobacter capsulatus* (*Rc*FPR) [4]. Such differences may be associated with their distinct C-terminal regions, where the *Rc*FPR has two additional residues (Figure S1). Thus, based on differences in the primary sequences in the C-terminal domains, a subdivision of subclass I bacterial FPRs into IA and IB has been proposed [4]. We here report the crystal structure of *Xac*FPR at a resolution of 1.5 Å. Even though *Xac*FPR adopts many structural characteristics of this bacterial subclass [4], loops distributed in the two domains show different mobility in terms of B factor values among FPRs from subclass IA and IB. Subclass IA FPRs, to which *Xac*FPR belongs, are characterised by the short C-terminal sequence VEK (Figure S1). The C-terminal glutamic residue side chain (Glu258, numbering as in *Xac*FPR) establishes an interaction with the O2′ of the FAD ribityl in the *Xac*FPR and *Pseudomonas aeruginosa* FPR (*Pa*FPR) structures, but not in that of *A. vinelandii *FPR (*Av*FPR). Finally, in *Xac*FPR, the exposure of FAD to the solvent is limited by interaction of the phosphoadenosine phosphate moiety of the cofactor with the side chain of Lys259, probably introducing structural restrictions to the enzyme catalytic turnover.

## 2. Materials and Methods

### 2.1. Protein Expression and Purification

*Xac*FPR was cloned and purified as previously reported [4]. Briefly, the coding sequence for *Xac*FPR, amplified by PCR, was introduced into pET28a vector between *Nde*I and *Eco*RI sites. The resulting 6His.tag-FPR fusion protein was purified using Ni-NTA chromatography, and, after thrombin treatment, a second affinity Ni-NTA chromatography separates the 6His.tag extension from the *Xac*FPR protein. Crystallization trials of *Xac*FPR demanded an additional purification step that consisted of a molecular exclusion chromatography using a Sephacryl S-200 column from GE Healthcare. The protein was obtained in 50 mM Tris-HCl pH 8.0, 150 mM NaCl and concentrated up to 20 mg/mL. For protein concentration calculation by absorbance spectra, a value of 10.7 mM^−1^ cm^−1^ for the extinction coefficient at 450 nm was used. 

### 2.2. Crystallization and Data Collection

Crystals of the *Xac*FPR were obtained using the hanging drop vapour diffusion method at 292 K. A typical drop contained 2 *μ*L of 20 mg/mL protein solution buffered with 50 mM Tris-HCl pH 8.0, 150 mM NaCl, and 4 *μ*L of reservoir solution containing 4.3 M NaCl and 100 mM Hepes pH 7.5. Droplets were equilibrated against 0.5 mL of reservoir solution. Crystals reached their maximum size in one week and were soaked in a cryoprotectant solution containing 75% of mother liquor and 25% of glycerol. Data were collected from a single crystal of *Xac*FPR using the synchrotron source ESRF beamline BM16 and an ADSC Quantum 210 detector with a wavelength of 0.99 Å, to a maximum resolution of 1.5 Å. The crystal belonged to the I222 orthorhombic space group with unit cell dimensions shown in Table 1. The calculated Matthews coefficient and the solvent content were 2.58 Å^3^/Da and 52.3%, respectively, corresponding to one molecule in the asymmetric unit. The data were processed and scaled using XDS [5] and SCALA [6] from the CCP4 package [7]. The structure was solved by molecular replacement using the program MOLREP from CCP4 and the structure of *Rc*FPR (PDB code 2BGJ) as the search model. The first model of the *Xac*FPR was automatically built with ARP/wARP [8]. Refinement was performed by REFMAC [9] from the CCP4 package, using the restrained refinement with atomic isotropic B factor and alternating manual model building by COOT [10]. The final model included residues 4–259, one FAD molecule, one Cl^−^ ion, and solvent molecules. Stereochemistry of the model was checked with PROCHECK [11]. MOLPROBITY [12] was used to assess the quality of the final structure. Two residues (T47 and E165) are in outliers region of Ramachandran plot, the electron density map around them being not good enough to position them precisely. Relevant data collection statistics and refinement parameters are presented in Table 1. The coordinates and structure factors for *Xac*FPR have been deposited in the Protein Data Bank with accession code 4B4D.

## 3. Results and Discussion

### 3.1. Overall Structure

**Figure 1:**
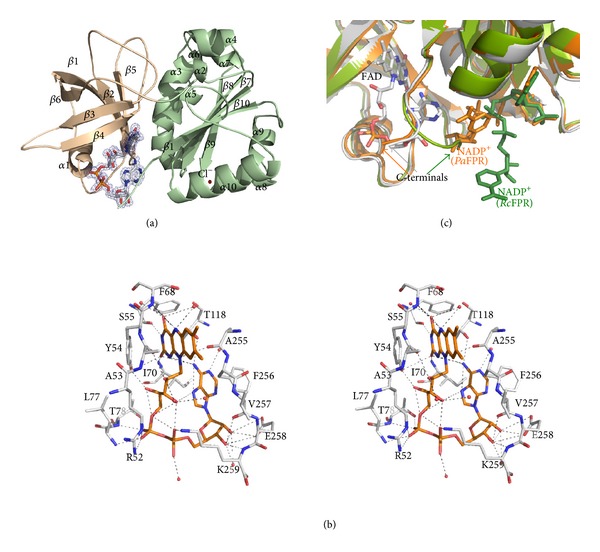
(a) Cartoon representation of *Xac*FPR structure. The N-domain is shown in yellow and the C-domain in green. The unbiased (before inclusion of FAD) |*F*_o_ | − | *F*_c_ | , *F*_calc_ electron density map is shown at 2.2 *σ*. FAD is in sticks with carbons in grey. Chloride ion is a red sphere. (b) Stereo representation of the FAD environment. Carbon atoms of amino acid residues and FAD are shown in white and orange, respectively. H-bonds involved in FAD stabilization are represented as dotted lines. (c) Cartoon superimposition of the active site of *Xac*FPR (in grey), *Pa*FPR:NADP^+^ complex (pdb 3crz, in orange), and *Rc*FPR:NADP^+^ complex (pdb 2vnj, in green). FAD, in sticks and with carbons in grey, belongs to *Xac*FPR structure and occupies similar position to FADs of *Pa* and *Rc*FPRs complexes. NADP^+^s from complexes are in sticks. C-terminuses of the three structures are pointed to by arrows.

The overall structure of *Xac*FPR strongly resembles those of bacterial FPRs from subclass I. It is organised in the two typical domains present in the ferredoxin-NADP(H) reductase family (Figure 1(a)). The N-terminus (residues 4–98) folds into a six-stranded antiparallel *β*-barrel (*β*1–*β*6) capped by helix *α*1. FAD binds to this domain, near the N-terminal end of *α*1 and the carboxy ends of the strands *β*4 and *β*5. The C-terminus (residues 99–259) adopts a classical nucleotide binding fold with a five-stranded parallel *β*-sheet surrounded by nine *α*-helices. The interface between both domains is formed by helix *α*3 of the C-domain and residues from loops connecting *β*2 with *β*3 and *β*4 with *β*5 at the N-domain. Finally, a chloride ion coming from the crystallization mother liquor is located between helices *α*8 and *α*10, at the C-domain, stabilised by the OG1 atom of Thr193 and two water molecules.

### 3.2. FAD Binding Site

Figure 1(b) shows the folded conformation and local environment of FAD in *Xac*FPR. As in all known bacterial FPR structures, the cofactor adopts a bent conformation that differs from the extended one in plastidic FNRs, placing the adenine and isoalloxazine rings in close proximity. The main driving force maintaining this bent conformation appears to be provided by the stacking of the adenine ring against the Phe256 and Ile70 side chains, while additional electrostatic and hydrophobic interactions contribute to stabilizing the overall binding. The O4 and O2 atoms of the FAD isoalloxazine ring interact with the amides of Ser55 and Ile70, respectively, while the N3 and N10 positions are stabilised by the backbone O atoms of Phe68, Ala53, and Ala255. The conserved Tyr54 is stacked in the *si*-face of FAD. Finally, the two C-terminal residues, Glu258 and Lys259, stabilise the position of the ribityl moiety of the cofactor through polar interactions, while the side chain of Lys259 establishes a salt bridge interaction with one of the phosphates of FAD (as reported for homologous Lys of *Pa*FPR and *Av*FPR). 

Based on the overall structure and FAD binding site organisation, *Xac*FPR seems to share many structural characteristics of other known bacterial FPRs. 

### 3.3. Comparison with Other Bacterial Subclass I FPRs

**Figure 2:**
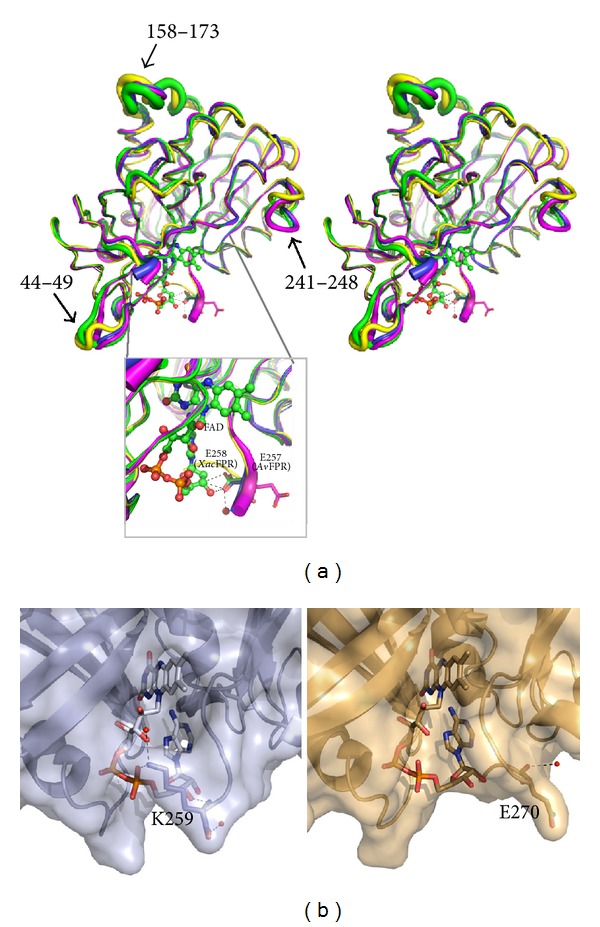
(a) Stereo cartoon representation of the *α*-carbon B-factors alignment for *Xac*FPR (green), *Av*FPR (pink), *Pa*FPR (blue) and *Rc*FPR (yellow) by Pymol. Positions with larger fluctuations are indicated in wide cartoon while those with the lower fluctuations are shown as thin cartoon. FAD from *Xac*FPR is displayed in space filling with carbons in green. Inset; detail of C-terminal homologous residues of FPRs from subclass IA in sticks. Interactions between Glu258 and FAD in *Xac*FPR are shown as dashed lines. The FAD and the side-chain of Glu258 are in space filling and sticks, respectively, and both CPK coloured. (b) Detail of the different exposition of FAD in *Xac*FPR (blue) and *Rc*FPR (dark yellow) due to the conformation of side chains of Lys259 and Glu270, respectively. Red balls represent water molecules and respective polar contacts of Lys259 and Glu270 with their environment are shown in dashed lines.

The crystal structure is quite similar to those of other subclass IA FPRs, *Pa*FPR, and *Av*FPR. Superposition of those structures onto that of *Xac*FPR shows r.m.s.d. values ~0.63 Å (for 227 and 237 C*α* atoms aligned, resp.) (Figure 2(a)), consistent with the high identity in structure-based sequence alignment (identity of 41.7% and 41%, resp.) (Figure S1). The third known structure of subclass I bacterial FPR, *Rc*FPR belonging to subclass IB, shows a slight higher r.m.s.d. value, 0.80 Å, when superposed to *Xac*FPR. The mayor overall difference among all these structures is in the conformation adopted for some residues in helices *α*6 and *α*7 and for the loop connecting them (residues 158–173, *Xac*FPR numbering) (Figure 2(a)). This region shows the highest B factor values in the* Rc*FPR and *Xac*FPR structures and, therefore, seems to present high flexibility in these FPRs (Figure 2(b)). This portion of the enzyme is situated in the protein surface opposite to the FAD binding site and also far away from the putative NADPH/NADP^+^ binding site. 

Two other long loops also differ in conformation with respect to the other subclass I FPRs: loop 44–49 that connects *β*3 with *β*4 and loop 241–248 located between *α*10 and *β*11. This latter loop, 241–248, exhibits low B factor values in *Xac*FPR, while they are relatively high in *Rc*FPR structure and moderate in *Av*FPR and *Pa*FPR. All those areas are well exposed to the solvent. It is not clear whether such differences are related to the reactivity towards the NAPDH coenzyme or to the interactions with their protein partners. 

The conformation of the last seven C-terminal residues of *Xac*FPR is identical to that of the equivalent residues *Pa*FPR and *Av*FPR, with the exception of the side chain of Glu258 (numbering *Xac*FPR) (Figure 2(a) inset). In *Xac*FPR structure, this residue adopts the same conformation as Glu257 in *Pa*FPR, pointing its OE1 side chain to the O2 ribityl of the FAD. In contrast, in *Av*FPR the corresponding amino acid is not engaged in such interaction and appears exposed to the solvent. The interaction of the C-terminal Lys259 with the phosphate of the FAD is conserved in *Pa*FPR and *Av*FPR (Figure 1(b)). This Lys residue is well conserved among all FPRs from subclass IA with the exception of *Neisseria meningitidis *(UniProt NMB1044) and *Xylella fastidiosa* (UniProt Q3R3Y4), where a Gln might play the Lys role. As a consequence of this interaction a decrease in mobility of the adenosine of FAD might be expected in subclass IA FPRs. 

The C-terminal extension in subclass I FPRs appears to be a mechanism to protect the expected stacking between the nicotinamide ring of the incoming hydride donor, NADPH, and the isoalloxazine of FAD, an interaction required for hydride transfer during catalysis. Therefore, a relatively large conformational rearrangement should take place in this region to achieve a catalytically competent interaction. All the FPR:NADP^+^ complexes for which structures have been so far solved show an apparent unproductive coenzyme binding (2vnh, 2vnj, 2vnk, 3crz) (Figure 1(c)). In the subclass IB *Rc*FPR:NADP^+^ complex, the nicotinamide hydride donor protrudes towards the solvent, far from producing a competent structure for hydride transfer [13]. The C-terminal end of the protein prevents the nicotinamide from entering into the active site, since the flavin *re*-face is closed by Ile272 and Arg158. Nevertheless, in the subclass IA *Pa*FPR:NADP^+^ complex [14] the shorter C-terminus apparently allows the nicotinamide to adopt a conformation that might precede the productive one (Figure 1(c)). This structural context in subclasses IA and IB FPRs could help to explain why *Xac*FPR, with the same conformation in its C-terminal residues as *Pa*FPR, exhibits higher catalytic efficiency in the NADPH-diaphorase activity than *Rc*FPR [4, 13] (Table S1). Moreover, while in the *Rc*FPR structure the side chain of Glu270 is orientated outwards of the structure, in *Xac*FPR, as mentioned previously, the homologous residue Lys259 binds the phosphate of FAD with its side chain (Figure 2(b)). This structural difference at C-terminus means a different exposure of the cofactor in both FPRs that might further contribute to explaining the different kinetic behaviours (Table S1). 

The structural information also allows postulating that in bacterial FPRs opening of the catalytic site for productive nicotinamide binding and allocation in the active site might take place through the interaction between the carboxylate groups of C-terminal residues (Lys259 in *Xac*FPR and Ile272 in *Rc*FPR) or the protein backbone and the nicotinamide of NADPH. Molecular dynamics simulations [15] recently identified the importance of the Tyr side chain of the C-terminal residue in plastidic FNRs (Y303 in *Anabaena* FNR) to obtain the optimum geometry for the hydride transfer process. In subclass I bacterial FPRs no equivalent residue to this plastidic Tyr is present, but the protein core formed by residues Ala255-Phe256-Val257 and the adenine moiety of the FAD in bent conformation might contribute in a similar manner. In that case, allocation of the nicotinamide in the active site in bacterial FPRs might require additional conformational changes of that region regarding the plastidic enzymes to obtain the catalytically competent complex. This could further contribute to explaining the low efficiency of this FPR as reductase as compared with plastidic enzymes.

## 4. Conclusions

Here we describe the first crystal structure of *Xac*FPR. This structure shows structural features that correspond to a protein from bacterial subclass IA but with detectable differences in the mobility of specific loops when compared with other known bacterial FPRs. Interactions involving the C-terminal Glu-Lys residues of *Xac*FPR with FAD are suggested to limit cofactor mobility, which might be a key point in defining the catalytic behaviour of the enzyme. Since *Xac*FPR is essential for *X. axonopodis* pv. citri survival, this structure constitutes a starting point in the development of drugs against citrus canker. 

## Supplementary Material

Table S1 with kinetic parameters of NADPH diaphorase reaction and dissociation constants for complexes with NADP^+^ for several plastidic and bacterial FNRs and Figure S1 with a structure-based sequence alignment of several bacterial FPR

## Figures and Tables

**Table 1:** Data collection and structural refinement statistics of *Xac*FPR.

Data collection statistics	
Space group	I222
Cell dimensions *a*, *b*, *c* (Å)	50.70, 99.78, 118.79
Wavelength, Å	0.99994
Resolution, Å	24.06–1.50 (1.58–1.50)
Total number of reflections	215020
Number of unique reflections	46901 (5597)
Redundancy	4.6 (2.9)
Completeness, %	96.7 (79.8)
Average *I*/*σ*	13.7 (1.9)
*R*_merge_^a^	0.057 (0.607)

Refinement statistics	
Resolution range, Å	24.06–1.50
Protein nonhydrogen atoms	2072
Ligand nonhydrogen atoms	54
Solvent nonhydrogen atoms	238
*R*_work_^b^ (%)	20.6
*R*_free_^b^ (%)	22.7
r.m.s.d. bond length, Å	0.007
r.m.s.d. bond angles, °	1.316
Average B factor, Å^2^	
All	21.60
Protein	20.98
Ligands: FAD Cl^−^	14.25 24.28
Waters	28.59

Ramachandran statistics	
Res. in preferred regions (%)	97.64
Res. in allowed regions (%)	1.57
Res. outliers (%)	0.79

Values in parentheses correspond to the highest resolution shell.

^a^ *R*_sym_ = ∑|*I* − *I*_av_|/∑*I*, where the summation is over symmetry equivalent reflections.

^b^ *R*_work_ and *R*_free_were calculated by the equation ∑||*F*_obs_| − |*F*_calc_||/∑|*F*_obs_|, where *F*_obs_ and *F*_calc_ are the observed and calculated structure factors, respectively. *R*_free_ was calculated for 7% of data excluded from the refinement.
