# Supplementary material for: Crystal Structure of the FAD-Containing Ferredoxin-NADP+ Reductase from the Plant Pathogen Xanthomonas axonopodis pv. citri
Source: Biomed Res Int. 2013 Aug 1;2013:906572. doi: 10.1155/2013/906572 (PMC3747371; doi:10.1155/2013/906572)
Supplement: Supplementary file 1 — Table S1 with kinetic parameters of NADPH diaphorase reaction and dissociation constants for complexes with NADP+ for several plastidic and bacterial FNRs and Figure S1 with a structure-based sequence alignment of several bacterial FPR [file 906572.f1.pdf]

Table S1. Kinetic parameters of NADPH diaphorase reaction for several FNRs and the dissociation constants for complexes with $NADP^+$

FNR	K_m (μM)	k_{cat} (s^{-1})	k_{cat}/K_m ($\mu M^{-1} s^{-1}$)	K_d (μM)
X. axonopodis[a]	10.8±0.5	121.9±1.8	11.3	9.7±0.4
pea[a]	15.3±4.3	374.3±18	24.5	10.9±3.1
E.coli[a]	8.3±1.3	38.2±3.5	4.6	5.9±0.6
R. capsulatus	80[b]	7.2 [b]	0.09	222±5[c]
P. putida[d]	4.79±0.08<br>3.97±0.04	4.77±0.17<br>4.510.06	1.03±0.02<br>1.14±0.08	-<br>-

[a] Data from Tondo M.L. et al., PLoS One 2011; 6: e27124.
[b] Data from Bittel C. et al., FEBS Lett 2003; 553: 408–412.
[c] Data from Bortolotti A. et al., BBA 2009; 1794: 199-210.
[d] Data from Yeom J. et al., J. Biochem 2009; 145: 481-491. Data for FprA (first line), homologue with the *A. vinelandii* FPR and FprB (second line), homologue with the *E.coli* FPR.

Figure S1

```
                         β1         β2              β3           β4
                                                                 ****
XacFP (4b4d)    -AFGAETVLEVRHWTDAYFSFTTTRDAGFRFENGQFVMIGLETET-RPLLRAYSIASANW 61
PaFPR (2qdx)    sNLYTERVLSVHHWNDTLFSFKTTRNPGLRFKTGQFVMIGLEVDG-RPLMRAYSIASPNY 60
AvFPR (1a8p)    sNLNVERVLSVHHWNDTLFSFKTTRNPSLRFENGQFVMIGLEVDG-RPLMRAYSIASPNY 60
RcFPR (2bgi)    --PDAQTVTSVRHWTDTLFSFRVTRPQTLRFRSGEFVMIGLLDDNgKPIMRAYSIASPAW 73
StFPR (3fpk)    aDWVTGKVTKVQNWTDALFSLTVHAP-iNPFTAGQFTKLGLEIDG-ERVQRAYSYVNAPD 59
EcFPR (1fdr)    aDWVTGKVTKVQNWTDALFSLTVHAP-vLPFTAGQFTKLGLEI----RVQRAYSYVNSPD 59

                    β5         α1      β6            α2       β7     α3
                      * *      **                                    *
XacFPR(4b4d)    EEHLEFFSIKVPDGPLTSRLQHIQPGDKVLVGKKPTGTLLISDLHPGRNLYLLGTGTGLA 121
PaFPR (2qdx)    EEHLEFFSIKVPDGPLTSRLQHLKEGDELMVSRKPTGTLVHDDLLPGKHLYLLSTGTGMA 120
AvFPR (1a8p)    EEHLEFFSIKVQNGPLTSRLQHLKEGDELMVSRKPTGTLVTSDLLPGKHLYMLSTGTGLA 120
RcFPR (2bgi)    DEELEFYSIKVPDGPLTSRLQHIKVGEQIILRPKPVGTLVIDALLPGKRLWFLATGTGIA 133
StFPR (3fpk)    NPNLEFYLVTVPQGKLSPRLAALKPGDEVQVVSDASGFFVLDEVPDCETLWMLATGTAIG 119
EcFPR (1fdr)    NPDLEFYLVTVPDGKLSPRLAALKPGDEVQVVSEAAGFFVLDEVPHCETLWMLATGTAIG 119

                          α4       β8           α5          α6      α7    β9
XacFPR(4b4d)    PWLSIIKDPETYERFDKVILTQGVRFVQDL-AYRDYFERELPQHEFLGDLLREKLLYYPA 180
PaFPR (2qdx)    PFLSVIQDPETYERYEKVILVHGVRWVSEL-AYADFITKVLPEHEYFGDQVKEKLIYYPL 179
AvFPR (1a8p)    PFMSLIQDPEVYERFEKVVLIHGVRQVNEL-AYQQFITEHLPQSEYFGEAVKEKLIYYPT 179
RcFPR (2bgi)    PFASLMREPEAYEKFDEVIMMHACRTVAELeYGRQLVE-ALQEDPLIGELVEGKLKYYPT 192
StFPR (3fpk)    PYLSILQYGQDVARFKNLVLVHAARFAADL-SYLPLML-ELQQR------YEGKLRIQTV 171
EcFPR (1fdr)    PYLSILRLGKDLDRFKNLVLVHAARYAADL-SYLPLMQ-ELEKR------YEGKLRIQTV 169

                              α8       α9               β10        α10
XacFPR(4b4d)    VTRETF--ANQGRLTELMADGRMQQTLGLPTLDPANDRFMICGSPQMLADLRSLLDS-RG 237
PaFPR (2qdx)    VTREPF--RNQGRQTDLMRSGKLFEDIGLPPMNPQDDRAMICGSPSMLEETSAVLDS-FG 236
AvFPR (1a8p)    VTRESF--HNQGRLTDLMRSGKLFEDIGLPPINPQDDRAMICGSPSMLDESCEVLDG-FG 236
RcFPR (2bgi)    TTREEF--HHMGRITDNLASGKVFEDLGIAPMNPETDRAMVCGSLAFNVDVMKVLES-YG 249
StFPR (3fpk)    VSRENVpgSLTGRVPALIENGELEKAVGLP-MDKETSHVMLCGNPQMVRDTQQLLKEtRQ 230
EcFPR (1fdr)    VSRETAagSLTGRIPALIESGELESTIGLP-MNKETSHVMLCGNPQMVRDTQQLLKEtRQ 230

                           β11
                                *****
XacFPR(4b4d)    FQTSPRIGTPGHYVFERAFVEK-- 259
PaFPR (2qdx)    LKISPRMGEPGDYLIERAFVEK-- 258
AvFPR (1a8p)    LKISPRMGEPGDYLIERAFVEK-- 258
RcFPR (2bgi)    LREG-ANSEPREFVVEKAFVGEgi 272
StFPR (3fpk)    MTKHLR-rRPGHMTAEHYW----- 248
EcFPR (1fdr)    MTKHLR-rRPGHMTAEHYW----- 248
```

Figure S1: Structure-based sequence alignment of several bacterial FPRs by DALI Server. Secondary structure for *Xac*FPR stated by the SMM server. Pa, *P. aeruginosa* (subclass IA); Av, *A. vinelandii* (subclass IA); Rc, *R. capsulatus* (subclass IB); St, *Salmonella typhimurium* (subclass II); Ec, *E. coli* (subclass II). Residues of *Xac*FPR that interact with FAD are pointed with an asterisk on top.
